# Outcome Analysis of Dual Implant Osteosynthesis for Ipsilateral Proximal and Shaft Femur Fractures: Do We Need Cephalomedullary Nails?

**DOI:** 10.7759/cureus.16613

**Published:** 2021-07-25

**Authors:** Saurabh Singh, Achyut Ravi, Pankaj Kumar Maurya, Rishabh Surana, Alok Rai

**Affiliations:** 1 Orthopedic Surgery, Banaras Hindu University, Varanasi, IND

**Keywords:** dual implant, osteosynthesis, femur, functional outcome, union

## Abstract

Introduction

Most surgeons prefer a single implant for segmental proximal and diaphyseal femur fractures, although results are controversial and still no consensus for proper management is present. This prospective study analyses the functional and radiological outcome of managing 17 patients with ipsilateral shaft and proximal femur fractures by dual implant osteosynthesis at our center.

Methods

Over a two-year period, we managed 17 patients with a mean age of 35 years, with cancellous cannulated screws or dynamic hip screws for intracapsular femur fractures and improvised proximal femoral nail for extracapsular proximal femur fractures. Distal femoral locking plates or distal femoral nails were used for shaft femur fractures depending upon fracture morphology. The patients had a maximum follow-up of 18 months.

Results

A total of 80% of patients had a good functional outcome (using the Friedman-Wyman scoring system) while 60% had an excellent Harris Hip Score. The mean time taken for the bone union for proximal femur fractures was 4.75 months and for shaft femur fractures, it was 6 months.

Conclusion

We had a satisfactory functional and clinical outcome of managing these fractures with two implants, one focusing biomechanically on each fracture. This principle of dual implant osteosynthesis can reliably be used in such difficult fracture patterns and it negates the use of the single cephalomedullary nail for fixating both fractures.

## Introduction

The percentage of an additional proximal femur fracture accompanying a femoral shaft is approximately 1%-9% [1]. The mechanism of injury is often attributed to high-energy trauma transferring the force to the shaft as well as proximal femur [1]. Also, in such cases, there is a reported 10%-30% chance of missing the proximal femur fracture at initial evaluation [2,3]. Various treatment strategies have been recommended; some authors suggest fixation with single implant, commonly cephalomedullary nails, but have reported non-union at one of the fracture sites, mostly diaphyseal fracture, in some of the patients [4]. Other authors suggest prompt surgery with priority to be given to femoral neck fracture fixation while some others advocate for fixation of the femoral shaft fracture first for providing better control of the leg during the subsequent more challenging femoral neck reduction [5-7]. Nevertheless, there is presently no consensus regarding the optimal treatment of these fractures, as to, which fracture should be fixed first and what implant to be used [8]. This prospective study reports the functional and radiological outcome analysis of dual implant osteosynthesis in managing 17 patients with ipsilateral femur shaft and proximal femur fractures.

## Materials and methods

A total of 32 patients presented to our center with ipsilateral proximal femoral and concomitant diaphyseal femur fractures, out of whom five patients had open wound at fracture site, three patients had ipsilateral tibia fracture, three patients were aged more than 60 years, two patients were deemed surgically unfit due to head injury and were conservatively managed for the time being and two patients had implant in situ and hence were excluded.

Seventeen patients with ipsilateral shaft femur and proximal femur fractures were treated within a period of two years from 2017 to 2019 at our tertiary care center in North India. Patients were followed up at regular intervals of one, three, six years and half yearly afterwards with the maximum follow-up being 18 months. There were 13 male and 4 female patients and the mean age was 35 years. Inclusion criteria were (1) age 18 to 60 years, (2) acute trauma less than three weeks old and (3) ipsilateral proximal femur and shaft fractures. Exclusion criteria were (1) any other fractures in ipsilateral limb, (2) patients with soft tissue defects or open wound, (3) those with coagulopathy, (4) poliotic limb, (5) previously operated with or without implant in situ (6) and surgically unfit patients. All patients had a high-energy mechanism of injury ranging from road traffic accidents to falls from a height.

All patients were primarily managed following standard Advanced Trauma Life Support (ATLS) guidelines and X-rays of the injured limbs were taken along with trauma series X-rays of the chest, pelvis and cervical spine. CT scans were taken for intercondylar distal femur fractures and also for undisplaced intracapsular femur fractures if any suspicion arose regarding fracture morphology. All patients with ipsilateral proximal femur and shaft femur fractures were given proper splintage and skeletal traction and were hemodynamically stabilized. After attaining pre-anesthetic fitness, patients were taken to surgery. Both the fractures were treated in single sitting with the shaft or distal femur fracture being fixed first and later the intracapsular or extracapsular proximal fracture.

After spinal anesthesia, patients were placed on the fracture table and surgically prepped and draped. The distal femur fracture was fixed first using the distal femur locking plate using the standard lateral approach and shaft femur fractures were fixed using distal femoral nails via a closed retrograde approach. Fixation of the distal fracture first results into sufficient traction, rotational stability and proper control of reduction for the proximal fracture. Next, the intracapsular femur fracture was fixed using standard percutaneous approach with three cancellous cannulated screws in an inverted triangle fashion or with the dynamic hip screw using the direct open lateral approach. Extracapsular proximal femur fractures were fixed using proximal femur nails.

On the second postoperative day, dressing was checked and physiotherapy started in the form of ankle, quadriceps and knee motion exercises. Suture removal was done at two weeks. After four weeks, partial weight bearing was allowed with walker support and consecutively weight bearing was increased in accordance with the progress of bone healing. Bone union was defined as bridging callus and bony trabeculations across the fracture in three out of four cortices in follow up X-rays. Parameters assessed at regular follow-ups were the Harris Hip Score, and the time of bone union of both the fractures and functional outcome using the Friedman-Wyman Scoring system [9,10]. Complications during the course of treatment were also noted.

**Figure 1:**
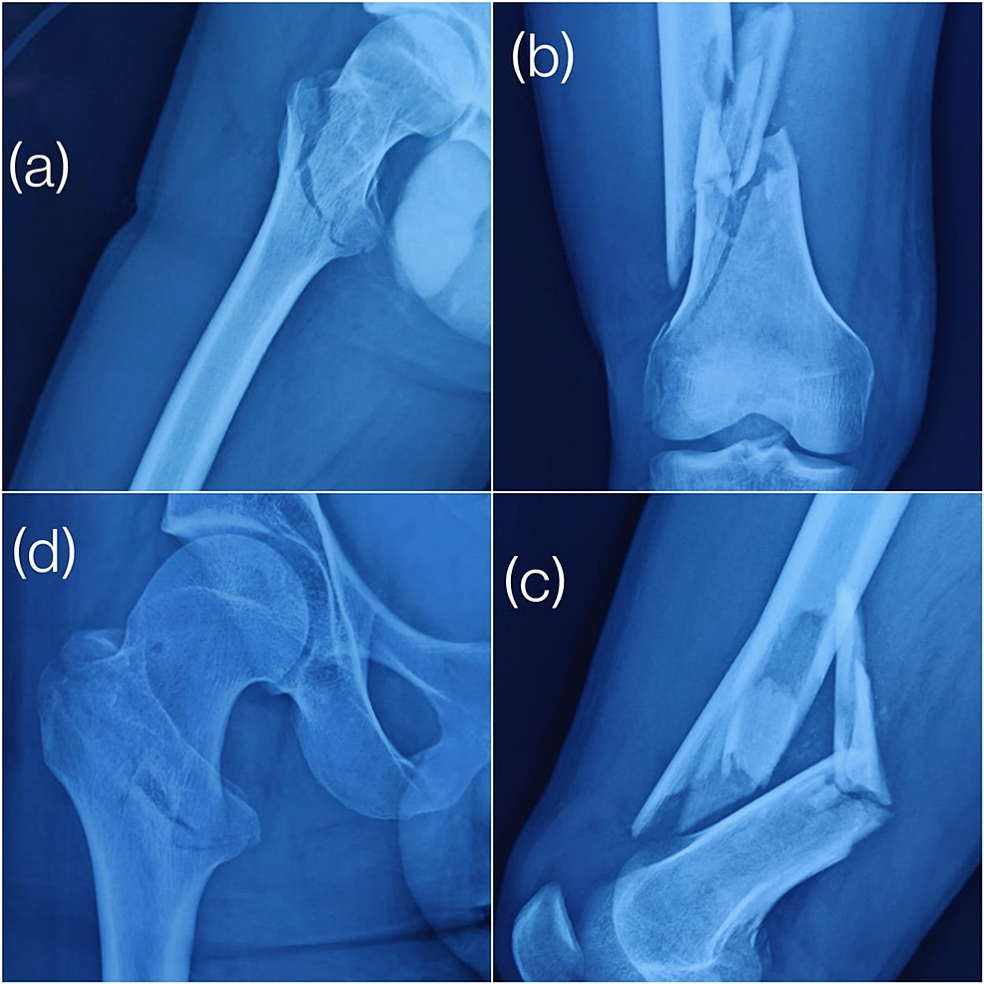
Pre-operative X-rays of a 38-year-old male (a) Lateral view of the proximal femur fracture; (b) antero-posterior view of the shaft femur fracture; (c) lateral view of the shaft femur fracture; (d) antero-posterior view of the proximal femur fracture

**Figure 2:**
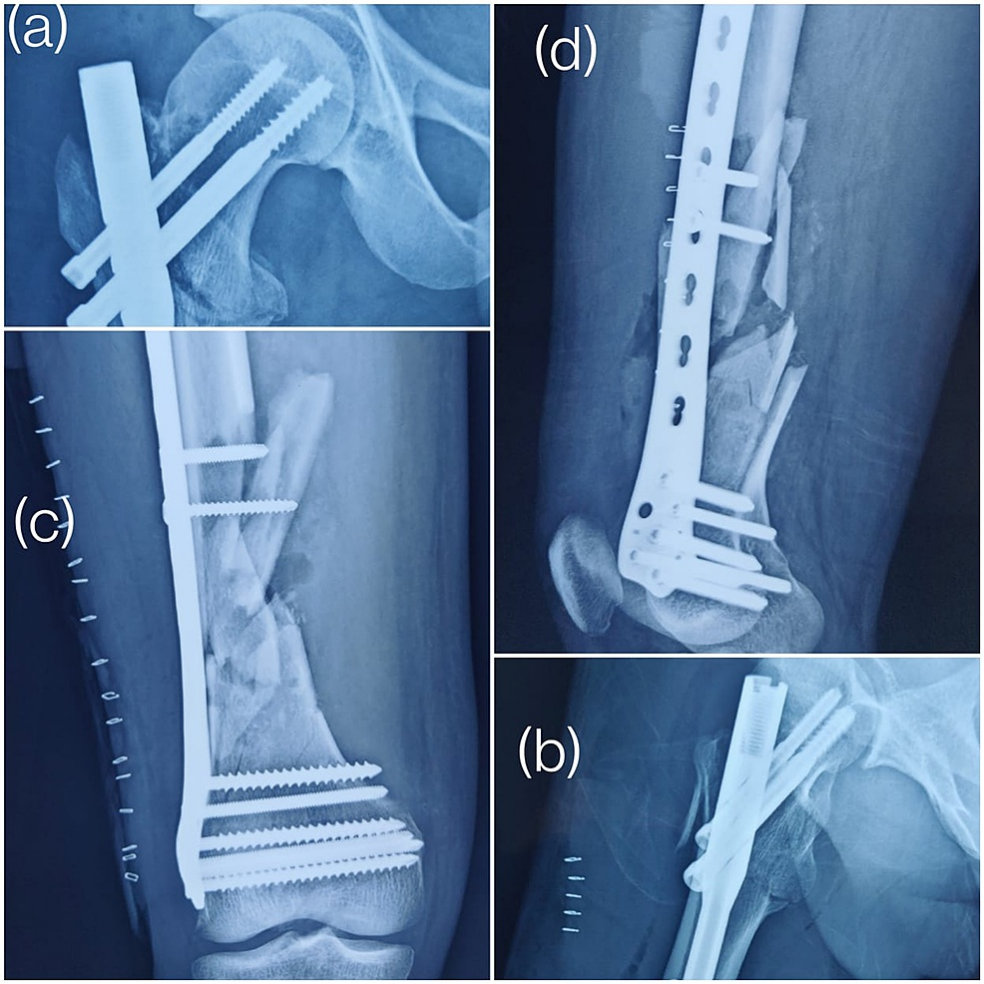
Post-operative day 1 X-rays Immediate post-operative X-rays showing (a) an antero-posterior view of fixation of the proximal femur fracture with a short proximal femoral nail; (b) a lateral view of fixation of the proximal femur fracture with a short proximal femoral nail; (c) an antero-posterior view of fixation of the shaft femur fracture with a distal femur locking plate; (d) a lateral view of fixation of the shaft femur fracture with a distal femur locking plate

Following is an example of a case: Figures 1a-1d show the pre-operative antero-posterior and lateral views of the proximal femur fracture and the shaft femur fracture. Figures 2a, 2b show the fixation of proximal femur fracture using the short proximal femoral nails and Figures 2c, 2d show fixation of the shaft femur fracture with the distal femur plate.

Statistical analysis was done using SPSS Statistics software (IBM Corp., Armonk, NY, USA). Significance was calculated based on Pearson’s chi-squared test. A result was considered to be statistically significant with a p-value <0.05. Written informed consent was taken from patients for publication of this study and any accompanying images.

## Results

Out of the total 17 patients with ipsilateral proximal and shaft femoral fractures, four patients had a combination of an intracapsular femur fracture with a distal femur fracture managed with cancellous cannulated screws and distal femoral locking plate, eight patients had extracapsular a femur fracture with a distal femur fracture managed with a short proximal femoral nail and distal femur locking plate, two patients had an intracapsular femur fracture along with a shaft femur fracture managed with cancellous cannulated screws and a distal femoral nail and three other patients had an extracapsular femur fracture with the shaft femur fracture managed with a short proximal femoral nail with a distal femoral nail.

Out of these 17 patients, 11 (64.7%) patients had an excellent Harris Hip Score at the final follow-up, 5 (29.4%) had good and 1 (5.8%) patient had a fair Harris Hip Score. Regarding the functional outcome measurement of these patients done by the Friedman-Wyman scoring system, 14 (82.3%) patients had a good functional outcome and 3 (17.6%) patients had a fair functional outcome as mentioned in Table 1.

**Table 1:** Details of individual patients with the final follow-up score DFLP = distal femoral locking plate; CCS = cancellous cannulated screw; sPFN = short proximal femoral nail; DFN = distal femoral nail; DHS = dynamic hip screw; M = male; F = female

S. no.	Age/sex	Diagnosis (fracture)	Implants used	Max. follow-up period (months)	Final follow-up			
					Harris Hip Score	Friedman-Wyman score	Time of union	
							Proximal femur	Shaft femur
1	36/M	Neck femur + Distal femur	CCS + DFLP	18	Excellent	Good	3 months	6 months
2	28/M	Neck femur + Distal femur	CCS + DFLP	18	Excellent	Good	3 months	9 months
3	30//M	Neck femur + Distal femur	CCS + DFLP	16	Good	Good	5 months	6 months
4	35/F	Intertrochanteric + Distal femur	sPFN + DFLP	15	Excellent	Good	4 months	7 months
5	40/M	Neck femur + Shaft femur	DHS + DFN	12	Excellent	Good	5 months	4 months
6	36/M	Intertrochanteric + Distal femur	sPFN + DFLP	13	Excellent	Good	7 months	9 months
7	27/F	Intertrochanteric + Shaft femur	sPFN + DFN	16	Excellent	Good	4 months	6 months
8	42/M	Neck femur + Distal femur	CCS + DFLP	15	Good	Fair	5 months	8 months
9	38/M	Intertrochanteric + Distal femur	sPFN + DFLP	18	Excellent	Good	4 months	7 months
10	35/F	Intertrochanteric + Distal femur	sPFN + DFLP	15	Excellent	Good	4 months	5 months
11	55/F	Intertrochanteric + Distal femur	sPFN + DFLP	15	Good	Fair	6 months	6 months
12	29/M	Intertrochanteric + Distal femur	sPFN + DFLP	16	Fair	Fair	7 months	6 months
13	35/M	Intertrochanteric + Shaft femur	sPFN + DFN	14	Good	Good	7 months	7 months
14	30/M	Intertrochanteric + Distal femur	sPFN + DFLP	14	Excellent	Good	7 months	9 months
15	38/M	Intertrochanteric + Distal femur	sPFN + DFLP	16	Excellent	Good	6 months	5 months
16	40/M	Intertrochanteric + Shaft femur	sPFN + DFN	15	Excellent	Good	6 months	4 months
17	35/M	Neck femur + Shaft femur	DHS + DFN	13	Good	Good	3 months	7 months

The intracapsular femur fracture was united in an average of four months with a range of three to six months. Extracapsular proximal fractures united in an average of five months with a range of four to seven months; diaphyseal fractures united in an average of 6.5 months with a range from four to nine months.

Two patients had superficial infections that resolved by antibiotics. Three patients had a delayed union at the distal femur fracture whereas their corresponding proximal fracture healed uneventfully, although none of the cases progressed into non-union. No case of osteonecrosis was seen at the final follow-up in the intracapsular fractures nor were any implant failures noted.

**Figure 3:**
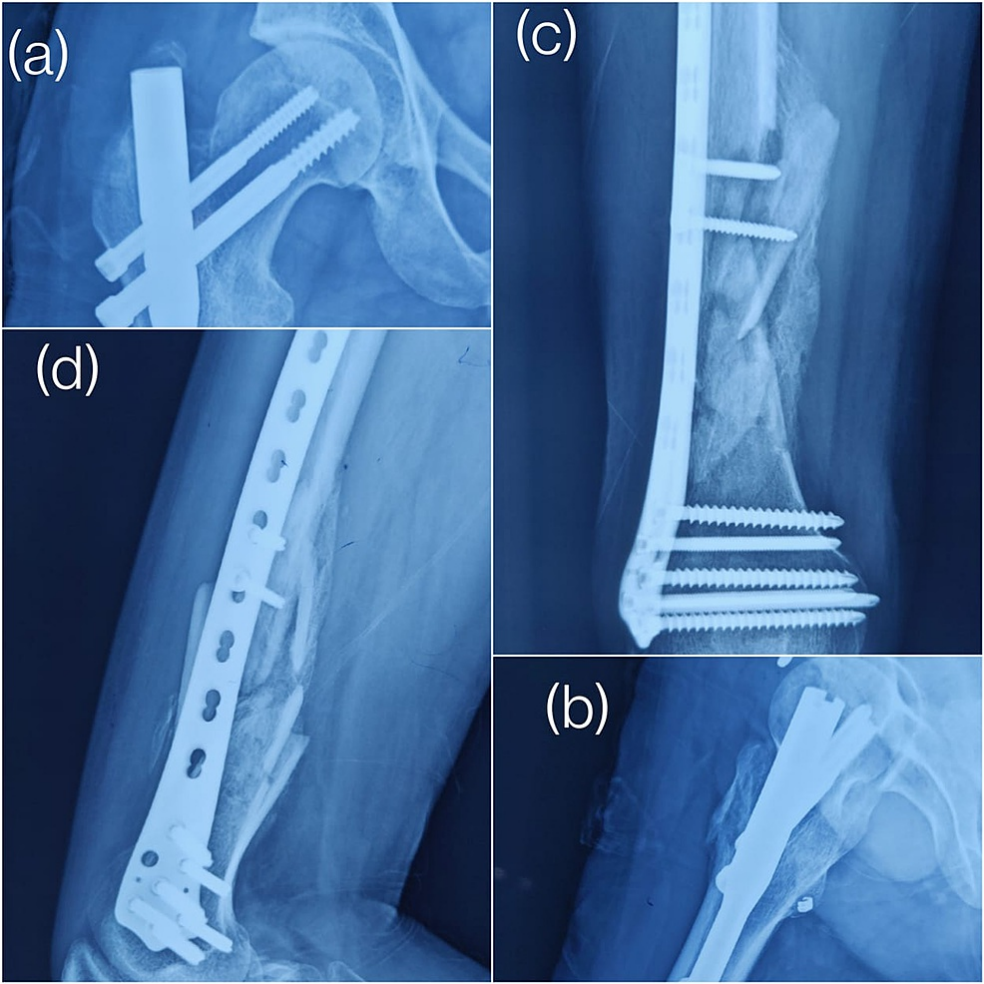
One-year follow-up X-rays (a) Antero-posterior and (b) lateral view of the union at the proximal femur fracture. (c) Antero-posterior and (d) lateral view of the union at the shaft femur fracture

Figure 3 shows the one-year follow-up X-ray of the aforementioned case showing union in the proximal femur fracture and union in the shaft femur fracture.

Table 2 shows the parameters used to determine the Friedman-Wyman score of these patients and Table 3 shows the individual score of all patients regarding these parameters. Table 4 shows the Harris Hip Score.

**Table 2:** Friedman-Wyman score

	Impaired daily life activity	Pain	Loss of hip and knee range of motion
Good	No	Nil	<20%
Fair	Mild	Mild-moderate	20%-50%
Poor	Moderate	Severe	>50%

**Table 3:** Friedman-Wyman scoring of individual patients M = male; F = female

S. no.	Age/sex	Impairment in daily life	Pain	Loss of range of motion
1	36/M	No impairment	Nil	15°
2	28/M	No impairment	Nil	10°
3	30//M	No impairment	Nil	10°
4	35/F	No impairment	Nil	0°
5	40/M	No impairment	Nil	0°
6	36/M	No impairment	Nil	5°
7	27/F	No impairment	Nil	5°
8	42/M	Mild impairment in activities	Mild	30°
9	38/M	No impairment	Nil	10°
10	35/F	No impairment	Nil	15°
11	55/F	Mild impairment in activities	Mild	30°
12	29/M	Mild impairment in activities	Moderate	40°
13	35/M	No impairment	Nil	5°
14	30/M	No impairment	Nil	10°
15	38/M	No impairment	Nil	15°
16	40/M	No impairment	Nil	5°
17	35/M	No impairment	Nil	10°

**Table 4:** Harris Hip Score

	Score
Excellent	90-100
Good	80-89
Fair	70-79
Poor	<70

**Figure 4:**
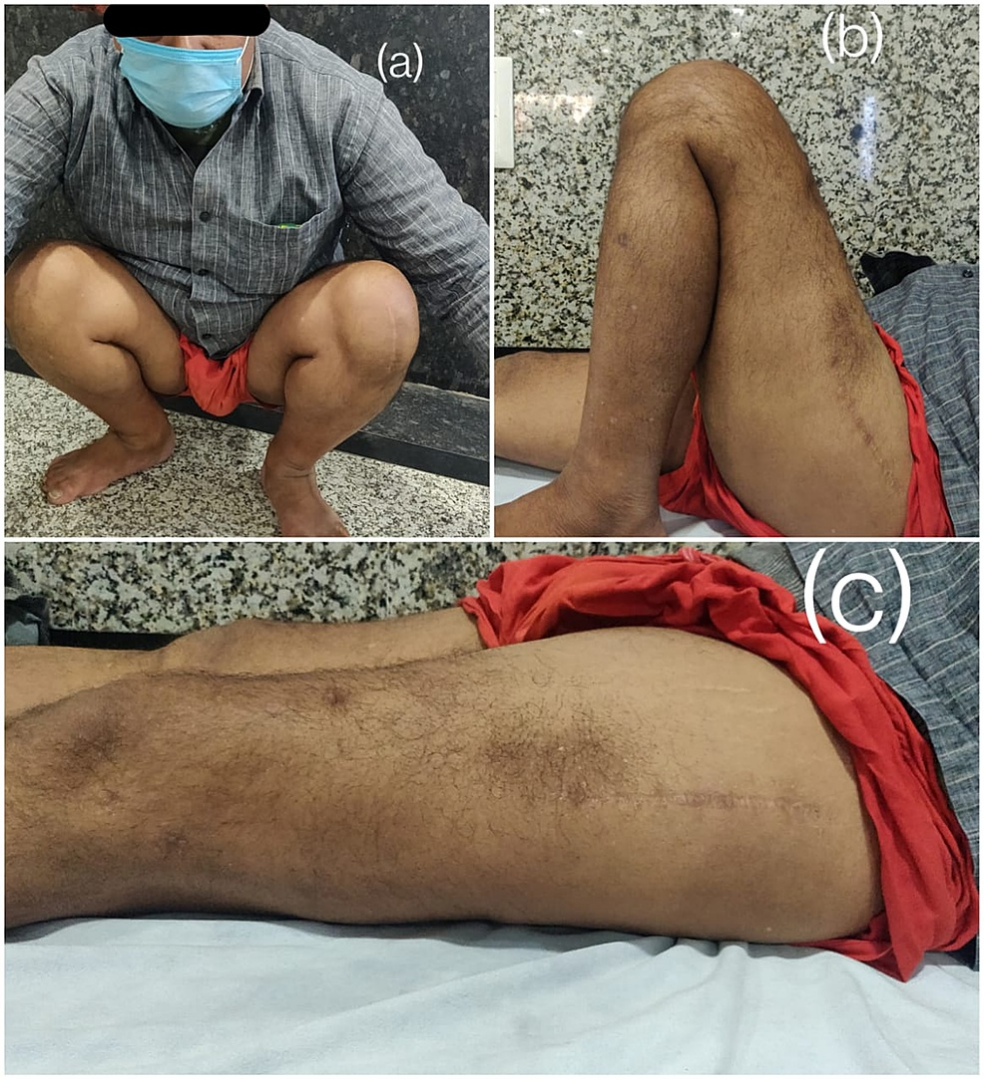
Clinical photographs showing the functional outcome at the one-year follow-up (a) Patient comfortably sitting in a squatting position; (b) flexion at knee; (c) extension at knee

Figure 4 shows the range of motion of the patient at the one-year follow-up.

## Discussion

Ipsilateral proximal femur and diaphyseal femur fractures are mostly seen as a result of high-energy trauma. Leung et al. reported 16 such cases with a mean age of 29 years [11]. Mohan et al. did a systematic review of six studies with 173 cases having an average age of 32 years [12]. Chaturvedi and Sahu and Abalo et al. with similar studies had a sample size of 17 and 37 cases with a mean age of 22-36 years and 37 years, respectively [13,14]. von Rüden et al. and Singh et al. did comparative studies over treatment of multilevel ipsilateral femoral fractures and studied 65 and 27 cases with an average age of 45 and 35 years, respectively [1,8]. We studied 17 patients whose mean age was around 35.8 years that indicates that the incidence of such fractures due to high-energy trauma is mostly in the third and fourth decades of life.

Most of the literature regarding this fracture has measured the functional outcome using the Friedman-Wyman score [9]. Mohan et al. in a systematic review of six studies compared the functional outcome of managing such fractures by single versus separate implants and 79.4% had a good outcome in the single implant group while 75% had a good functional outcome in fractures managed by separate implants [12]. In a similar study by Leung et al., functional outcome was measured similarly and 87.5% had a good outcome while 12.5% had a fair outcome [11]. Abalo et al. evaluated the outcome of managing ipsilateral femur neck and shaft fracture by the dynamic hip screw and dynamic compression plate and found 78% had good, 13.5% had fair and 8.5% had poor functional outcomes [14]. Singh et al. did a comparative study of managing multilevel ipsilateral shaft femoral fractures using two different methods: one group with the single intramedullary nail and other group with two plate combinations and found no significant differences in the functional outcome of both the groups [8]. Wang et al. in a similar comparative study compared one group of patients managed by long proximal femoral nail antirotation (PFNA) with another group managed by plate combinations and found no significant difference in the functional outcome of both the groups [15]. In our study of dual implant osteosynthesis in such fractures, we found 82.3% patients had a good functional outcome and 17.6% had a fair outcome.

Abalo et al. studied surgical outcomes in 37 patients with ipsilateral femur shaft and femur neck fractures managed by separate implants and found that 92% neck femur fractures united in an average of four months while 87% of shaft femur fractures united within six months [14]. Mohan et al. did a meta-analysis of six non-randomized cohort studies assessing 173 patients and found that due to lack of randomized studies, it was difficult to recommend a single or separate device treatment approach and that more prospective randomized studies were needed on the topic to come to a proper consensus [12]. Chaturvedi and Sahu treated 17 cases with concomitant fractures of the ipsilateral shaft and neck and the time of union for neck fractures was an average of four months and the time of union for shaft femoral fractures was an average of three months two weeks [13]. Singh et al. did a comparative study of managing multilevel ipsilateral shaft femoral fractures using two different methods: one group using a single intramedullary nail and other group with two plate combinations, and found no significant difference in both the groups regarding the time taken for bone union [8]. In our study, the average time of union of the proximal femur fracture was 5.05 months and the average time of union for the shaft femoral fracture was 6.5 months.

In our study, we first stabilized the distal shaft fracture and fixed the neck or intertrochanteric fracture afterwards. The limitations of the present study include the small number of patients and the potential for user bias, because the surgeon could not be blinded with respect to the method used. Also, the overall cost of surgery and the time for surgery increase as two implants have to be used. Two different incisions have to be used that is another limitation regarding this procedure. These limitations notwithstanding, this prospective study showed that this treatment method is a reliable option in the management of ipsilateral proximal femoral and shaft fractures.

## Conclusions

Dual implant osteosynthesis offers a biomechanically improved management of ipsilateral femur shaft and proximal femur fractures and addresses both fractures separately thus providing the bone union. Although we would like to encourage the use of dual implants for satisfactory functional and clinical outcome in the management of these types of fractures, we suggest more studies with longer follow-ups, multiple blinding, greater sample size, control groups and comparisons to come to a proper consensus to be followed methodically for such surgically challenging fractures.
